# 2-{(1*R*,2*R*)-2-[Bis(4-methyl­benz­yl)amino]­cyclo­hex­yl}isoindoline-1,3-dione

**DOI:** 10.1107/S1600536811018101

**Published:** 2011-05-20

**Authors:** Chao Li, Xiang-Kai Fu, Chuan-Long Wu, Jing Huang

**Affiliations:** a College of Chemistry and Chemical Engineering, Research Institute of Applied Chemistry, Southwest University, The Key Laboratory of Applied Chemistry of Chongqing Municipality, Chongqing 400715, People’s Republic of China

## Abstract

In the title mol­ecule, C_30_H_32_N_2_O_2_, the two tolyl rings form dihedral angles of 65.8 (1) and 6.6 (1)° with the isoindole-1,3-dione mean plane. The cyclo­hexane ring adopts a chair conformation.

## Related literature

For applications of chiral tertiary amines as catalysts for direct aldol reactions, see: Paradowska *et al.* (2009 ▶). For details of the synthesis, see: Kaik & Gawroński (2003 ▶); Gawronski *et al.* (1998 ▶).
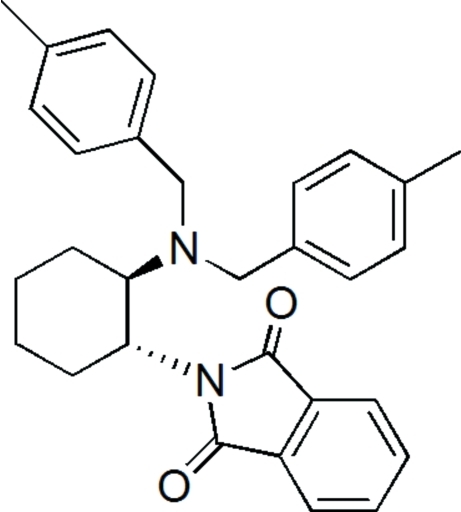


## Experimental

### 

#### Crystal data

C_30_H_32_N_2_O_2_
*M* *_r_* = 452.58
Monoclinic, 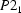

*a* = 12.472 (2) Å
*b* = 9.2853 (17) Å
*c* = 12.505 (2) Å
β = 115.305 (2)°
*V* = 1309.1 (4) Å^3^
*Z* = 2
Mo *K*α radiation
μ = 0.07 mm^−1^
*T* = 298 K
0.38 × 0.24 × 0.24 mm

#### Data collection

Bruker SMART APEX diffractometer
6901 measured reflections
2597 independent reflections
2042 reflections with *I* > 2σ(*I*)
*R*_int_ = 0.022

#### Refinement

*R*[*F* ^2^ > 2σ(*F* ^2^)] = 0.047
*wR*(*F* ^2^) = 0.112
*S* = 1.11
2597 reflections
309 parameters
1 restraint
H-atom parameters constrained
Δρ_max_ = 0.10 e Å^−3^
Δρ_min_ = −0.11 e Å^−3^

### 

Data collection: *SMART* (Bruker, 2000 ▶); cell refinement: *SAINT* (Bruker, 2000 ▶); data reduction: *SAINT*; program(s) used to solve structure: *SHELXS97* (Sheldrick, 2008 ▶); program(s) used to refine structure: *SHELXL97* (Sheldrick, 2008 ▶); molecular graphics: *SHELXTL* (Sheldrick, 2008 ▶); software used to prepare material for publication: *SHELXTL*.

## Supplementary Material

Crystal structure: contains datablocks I, global. DOI: 10.1107/S1600536811018101/cv5079sup1.cif

Structure factors: contains datablocks I. DOI: 10.1107/S1600536811018101/cv5079Isup2.hkl

Additional supplementary materials:  crystallographic information; 3D view; checkCIF report

## Figures and Tables

**Fig. 1.**
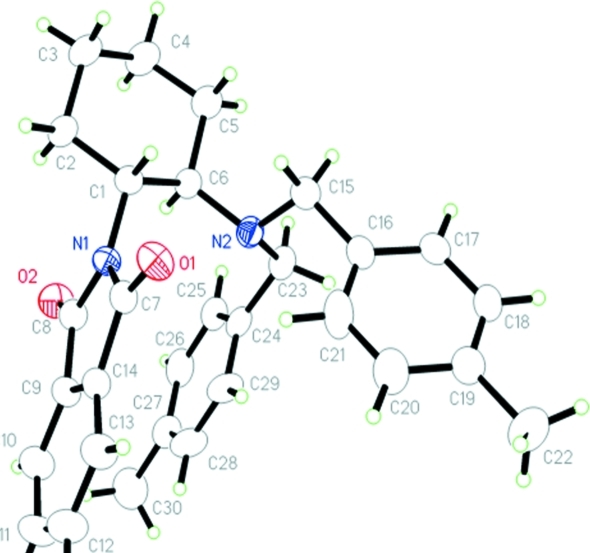
The molecular structure of (I) showing the atomic numbering and 20% probability displacement ellipsoids.

## supplementary crystallographic information

### Comment

Chiral tertiary amines are efficient catalysts for direct aldol reactions of ketones with aldehydes (Paradowska *et al.*, 2009). Therefore, it is of great interest for us to investigate the novel chiral tertiary amine as a chiral catalyst. In this article we would like to report the crystal structure of the title compound (I).

In (I) (Fig. 1), two tolyl rings form dihedral angles of 65.8 (1) and 6.6 (1)°, respectively, with the isoindole-1,3-dione mean plane. Cyclohexane ring adopts a chair conformation. This type of molecular geometry was reported also by Gawronski *et al.* (1998). It could be found that in the crystal structures the cyclohexane rings adopt chair conformations with phthalimide rings in equatorial orientation.

### Experimental

To a solution of (1*R*,2*R*)-*N*-phthaloyl-1,2-diaminocyclohexane (2.44 g, 10 mmol) (Kaik *et al.*, 2003; Gawronski *et al.*, 1998) in acetonitrile (50 ml) was added at room temperature K_2_CO_3_ (3.20 g, 23 mmol) and 4-methylbenzyl chloride (3 mL, 25 mmol). The mixture was refluxed with stirring for 5 h. The solvent was removed *in vacuo* and the mixture was extracted with dichloromethane and NaHCO_3_ solution. The organic solution was dried over MgSO_4_ and evaporated. Product was directly purified through flash column chromatography on a slilca gel to afford white solid. A crystal of (I) suitable for X-ray analysis was grown from diethyl ether by slow evaporation at room temperature.

### Refinement

All H atoms were placed in idealized positions and treated as riding, with C—H = 0.96 (CH_3_), *U_i_*_so_(H) = 1.5 *U*_eq_(CH_3_), and C—H = 0.97 (CH_2_), 0.98 or 0.93 Å (CH), *U_i_*_so_(H) = 1.2 *U*_eq_(CH and CH_2_). In the absence of any significant anomalous scatterers in the molecule, attempts to confirm the absolute structure by refinement of the Flack parameter in the presence of 1479 sets of Friedel equivalents led to an inconclusive value of -1.1 (17). Therefore, the Friedel pairs were merged before the final refinement and the absolute configuration was assigned to correspond with that of the known chiral centres in a precursor molecule, which remained unchanged during the synthesis of the title compound.

### Crystal data

**Table:**

C_30_H_32_N_2_O_2_	*F*(000) = 484
*M**_r_* = 452.58	*D*_x_ = 1.148 Mg m^−^^3^
Monoclinic, *P*2_1_	Mo *K*α radiation, λ = 0.71073 Å
Hall symbol: P 2yb	Cell parameters from 1969 reflections
*a* = 12.472 (2) Å	θ = 2.8–21.3°
*b* = 9.2853 (17) Å	µ = 0.07 mm^−^^1^
*c* = 12.505 (2) Å	*T* = 298 K
β = 115.305 (2)°	Block, colourless
*V* = 1309.1 (4) Å^3^	0.38 × 0.24 × 0.24 mm
*Z* = 2	

### Data collection

**Table:**

Bruker SMART APEX diffractometer	2042 reflections with *I* > 2σ(*I*)
Radiation source: fine-focus sealed tube	*R*_int_ = 0.022
graphite	θ_max_ = 25.5°, θ_min_ = 1.8°
φ and ω scans	*h* = −13→15
6901 measured reflections	*k* = −9→11
2597 independent reflections	*l* = −14→15

### Refinement

**Table:**

Refinement on *F*^2^	Primary atom site location: structure-invariant direct methods
Least-squares matrix: full	Secondary atom site location: difference Fourier map
*R*[*F*^2^ > 2σ(*F*^2^)] = 0.047	Hydrogen site location: inferred from neighbouring sites
*wR*(*F*^2^) = 0.112	H-atom parameters constrained
*S* = 1.11	*w* = 1/[σ^2^(*F*_o_^2^) + (0.0526*P*)^2^ + 0.0478*P*] where *P* = (*F*_o_^2^ + 2*F*_c_^2^)/3
2597 reflections	(Δ/σ)_max_ = 0.001
309 parameters	Δρ_max_ = 0.10 e Å^−^^3^
1 restraint	Δρ_min_ = −0.11 e Å^−^^3^

### Special details

**Table:**

Geometry. All e.s.d.'s (except the e.s.d. in the dihedral angle between two l.s. planes) are estimated using the full covariance matrix. The cell e.s.d.'s are taken into account individually in the estimation of e.s.d.'s in distances, angles and torsion angles; correlations between e.s.d.'s in cell parameters are only used when they are defined by crystal symmetry. An approximate (isotropic) treatment of cell e.s.d.'s is used for estimating e.s.d.'s involving l.s. planes.
Refinement. Refinement of *F*^2^ against ALL reflections. The weighted *R*-factor *wR* and goodness of fit *S* are based on *F*^2^, conventional *R*-factors *R* are based on *F*, with *F* set to zero for negative *F*^2^. The threshold expression of *F*^2^ > σ(*F*^2^) is used only for calculating *R*-factors(gt) *etc*. and is not relevant to the choice of reflections for refinement. *R*-factors based on *F*^2^ are statistically about twice as large as those based on *F*, and *R*- factors based on ALL data will be even larger.

### Fractional atomic coordinates and isotropic or equivalent isotropic displacement parameters (Å^2^)

**Table:**

	*x*	*y*	*z*	*U*_iso_*/*U*_eq_	
C1	0.3760 (3)	0.0454 (4)	0.7604 (3)	0.0615 (8)	
H1	0.3811	0.0357	0.8405	0.074*	
C2	0.4270 (3)	−0.0921 (4)	0.7338 (3)	0.0780 (10)	
H2A	0.4295	−0.0825	0.6577	0.094*	
H2B	0.5076	−0.1051	0.7934	0.094*	
C3	0.3546 (3)	−0.2234 (4)	0.7319 (4)	0.0917 (12)	
H3A	0.3862	−0.3069	0.7084	0.110*	
H3B	0.3606	−0.2408	0.8108	0.110*	
C4	0.2260 (3)	−0.2031 (4)	0.6470 (4)	0.1012 (13)	
H4A	0.1805	−0.2863	0.6503	0.121*	
H4B	0.2190	−0.1949	0.5669	0.121*	
C5	0.1767 (3)	−0.0685 (4)	0.6784 (4)	0.0910 (12)	
H5A	0.1791	−0.0802	0.7565	0.109*	
H5B	0.0944	−0.0568	0.6225	0.109*	
C6	0.2455 (3)	0.0666 (4)	0.6772 (3)	0.0658 (8)	
H6	0.2410	0.0744	0.5972	0.079*	
C7	0.4942 (2)	0.2658 (4)	0.8556 (2)	0.0581 (7)	
C8	0.4647 (3)	0.2219 (4)	0.6645 (3)	0.0641 (8)	
C9	0.5322 (3)	0.3581 (4)	0.7045 (2)	0.0646 (8)	
C10	0.5750 (3)	0.4513 (5)	0.6459 (3)	0.0876 (11)	
H10	0.5641	0.4332	0.5687	0.105*	
C11	0.6349 (3)	0.5730 (5)	0.7066 (4)	0.0955 (12)	
H11	0.6642	0.6386	0.6695	0.115*	
C12	0.6514 (3)	0.5976 (5)	0.8200 (4)	0.0961 (12)	
H12	0.6931	0.6790	0.8592	0.115*	
C13	0.6079 (3)	0.5051 (4)	0.8780 (3)	0.0806 (10)	
H13	0.6180	0.5237	0.9548	0.097*	
C14	0.5493 (2)	0.3844 (4)	0.8184 (2)	0.0588 (8)	
C15	0.1637 (3)	0.1925 (5)	0.7988 (3)	0.0837 (10)	
H15A	0.2246	0.1407	0.8638	0.100*	
H15B	0.0903	0.1388	0.7738	0.100*	
C16	0.1474 (3)	0.3400 (5)	0.8414 (3)	0.0747 (9)	
C17	0.0420 (4)	0.3825 (5)	0.8398 (4)	0.0908 (12)	
H17	−0.0231	0.3211	0.8086	0.109*	
C18	0.0302 (4)	0.5158 (5)	0.8841 (4)	0.0945 (13)	
H18	−0.0424	0.5407	0.8833	0.113*	
C19	0.1212 (4)	0.6106 (5)	0.9284 (3)	0.0828 (11)	
C20	0.2259 (4)	0.5699 (6)	0.9282 (4)	0.1076 (14)	
H20	0.2899	0.6331	0.9568	0.129*	
C21	0.2390 (4)	0.4372 (7)	0.8866 (4)	0.1091 (15)	
H21	0.3123	0.4124	0.8890	0.131*	
C22	0.1084 (4)	0.7562 (6)	0.9758 (4)	0.1147 (15)	
H22A	0.0400	0.7556	0.9925	0.172*	
H22B	0.0990	0.8290	0.9180	0.172*	
H22C	0.1779	0.7763	1.0471	0.172*	
C23	0.0991 (3)	0.2557 (5)	0.5925 (3)	0.0788 (10)	
H23A	0.0567	0.3291	0.6141	0.095*	
H23B	0.0443	0.1777	0.5543	0.095*	
C24	0.1419 (3)	0.3184 (4)	0.5070 (3)	0.0675 (9)	
C25	0.1056 (3)	0.2635 (5)	0.3942 (3)	0.0793 (10)	
H25	0.0565	0.1830	0.3707	0.095*	
C26	0.1426 (3)	0.3289 (6)	0.3161 (3)	0.0906 (13)	
H26	0.1169	0.2914	0.2402	0.109*	
C27	0.2150 (4)	0.4459 (5)	0.3467 (3)	0.0855 (11)	
C28	0.2522 (4)	0.4976 (5)	0.4595 (4)	0.0971 (12)	
H28	0.3027	0.5767	0.4833	0.117*	
C29	0.2161 (4)	0.4344 (4)	0.5382 (3)	0.0859 (11)	
H29	0.2429	0.4716	0.6143	0.103*	
C30	0.2538 (5)	0.5162 (8)	0.2598 (4)	0.140 (2)	
H30A	0.2079	0.4782	0.1820	0.209*	
H30B	0.3363	0.4967	0.2828	0.209*	
H30C	0.2417	0.6184	0.2593	0.209*	
N1	0.4460 (2)	0.1719 (3)	0.76050 (19)	0.0566 (6)	
N2	0.1976 (2)	0.2011 (3)	0.6999 (2)	0.0671 (7)	
O1	0.49097 (18)	0.2479 (3)	0.95003 (16)	0.0775 (7)	
O2	0.4310 (2)	0.1639 (3)	0.56978 (19)	0.0869 (7)	

### Atomic displacement parameters (Å^2^)

**Table:**

	*U*^11^	*U*^22^	*U*^33^	*U*^12^	*U*^13^	*U*^23^
C1	0.0628 (17)	0.062 (2)	0.0575 (16)	−0.0014 (16)	0.0233 (14)	0.0042 (15)
C2	0.0661 (19)	0.065 (2)	0.092 (2)	0.0084 (18)	0.0231 (18)	0.0055 (19)
C3	0.085 (2)	0.061 (2)	0.116 (3)	0.005 (2)	0.031 (2)	0.003 (2)
C4	0.084 (2)	0.066 (3)	0.132 (3)	−0.006 (2)	0.026 (2)	−0.014 (2)
C5	0.062 (2)	0.076 (3)	0.122 (3)	−0.008 (2)	0.026 (2)	−0.006 (2)
C6	0.0595 (17)	0.065 (2)	0.0681 (18)	0.0039 (17)	0.0226 (15)	−0.0002 (17)
C7	0.0476 (15)	0.072 (2)	0.0478 (15)	0.0018 (15)	0.0139 (12)	0.0009 (15)
C8	0.0733 (19)	0.070 (2)	0.0529 (17)	0.0020 (17)	0.0310 (15)	−0.0014 (17)
C9	0.0653 (18)	0.071 (2)	0.0595 (18)	−0.0003 (17)	0.0288 (15)	0.0083 (17)
C10	0.104 (3)	0.091 (3)	0.078 (2)	−0.009 (3)	0.049 (2)	0.007 (2)
C11	0.104 (3)	0.087 (3)	0.101 (3)	−0.024 (3)	0.048 (2)	0.011 (3)
C12	0.091 (3)	0.081 (3)	0.098 (3)	−0.020 (2)	0.023 (2)	0.000 (2)
C13	0.081 (2)	0.086 (3)	0.067 (2)	−0.015 (2)	0.0242 (18)	−0.008 (2)
C14	0.0514 (16)	0.065 (2)	0.0538 (16)	−0.0007 (15)	0.0161 (13)	−0.0004 (15)
C15	0.093 (2)	0.080 (3)	0.093 (2)	0.007 (2)	0.053 (2)	0.010 (2)
C16	0.081 (2)	0.084 (3)	0.0734 (19)	0.006 (2)	0.0466 (18)	0.009 (2)
C17	0.095 (3)	0.080 (3)	0.120 (3)	−0.003 (2)	0.067 (2)	0.010 (3)
C18	0.097 (3)	0.093 (3)	0.118 (3)	0.016 (3)	0.071 (3)	0.014 (3)
C19	0.098 (3)	0.094 (3)	0.063 (2)	0.005 (2)	0.039 (2)	0.004 (2)
C20	0.092 (3)	0.125 (4)	0.104 (3)	−0.015 (3)	0.041 (2)	−0.038 (3)
C21	0.079 (2)	0.140 (4)	0.115 (3)	0.003 (3)	0.048 (2)	−0.037 (3)
C22	0.150 (4)	0.103 (4)	0.087 (3)	0.015 (3)	0.048 (3)	−0.009 (3)
C23	0.0663 (19)	0.084 (3)	0.079 (2)	0.016 (2)	0.0249 (17)	0.001 (2)
C24	0.0640 (18)	0.064 (2)	0.0644 (19)	0.0193 (17)	0.0176 (15)	0.0024 (17)
C25	0.0636 (19)	0.085 (3)	0.078 (2)	0.0056 (19)	0.0194 (17)	−0.015 (2)
C26	0.080 (2)	0.119 (4)	0.062 (2)	0.018 (3)	0.0195 (19)	−0.012 (2)
C27	0.090 (3)	0.089 (3)	0.074 (2)	0.022 (3)	0.031 (2)	0.016 (2)
C28	0.119 (3)	0.066 (2)	0.091 (3)	−0.001 (2)	0.031 (2)	0.009 (2)
C29	0.120 (3)	0.063 (2)	0.064 (2)	0.003 (2)	0.029 (2)	0.0004 (19)
C30	0.149 (4)	0.164 (6)	0.126 (4)	0.026 (4)	0.078 (3)	0.040 (4)
N1	0.0595 (13)	0.0603 (16)	0.0502 (12)	−0.0025 (13)	0.0236 (11)	0.0015 (12)
N2	0.0656 (15)	0.0680 (19)	0.0686 (15)	0.0087 (14)	0.0296 (13)	0.0038 (14)
O1	0.0797 (13)	0.1028 (19)	0.0471 (11)	−0.0103 (14)	0.0243 (10)	0.0023 (12)
O2	0.1193 (18)	0.0876 (17)	0.0625 (13)	−0.0082 (16)	0.0472 (13)	−0.0137 (13)

### Geometric parameters (Å, °)

**Table:**

C1—N1	1.463 (4)	C15—C16	1.514 (6)
C1—C6	1.524 (4)	C15—H15A	0.9700
C1—C2	1.525 (4)	C15—H15B	0.9700
C1—H1	0.9800	C16—C17	1.365 (5)
C2—C3	1.511 (5)	C16—C21	1.374 (6)
C2—H2A	0.9700	C17—C18	1.390 (6)
C2—H2B	0.9700	C17—H17	0.9300
C3—C4	1.510 (5)	C18—C19	1.354 (6)
C3—H3A	0.9700	C18—H18	0.9300
C3—H3B	0.9700	C19—C20	1.360 (6)
C4—C5	1.517 (6)	C19—C22	1.512 (6)
C4—H4A	0.9700	C20—C21	1.374 (7)
C4—H4B	0.9700	C20—H20	0.9300
C5—C6	1.524 (5)	C21—H21	0.9300
C5—H5A	0.9700	C22—H22A	0.9600
C5—H5B	0.9700	C22—H22B	0.9600
C6—N2	1.464 (4)	C22—H22C	0.9600
C6—H6	0.9800	C23—N2	1.470 (4)
C7—O1	1.211 (3)	C23—C24	1.501 (5)
C7—N1	1.387 (4)	C23—H23A	0.9700
C7—C14	1.474 (4)	C23—H23B	0.9700
C8—O2	1.201 (4)	C24—C29	1.364 (5)
C8—N1	1.398 (4)	C24—C25	1.382 (4)
C8—C9	1.483 (5)	C25—C26	1.385 (5)
C9—C14	1.369 (4)	C25—H25	0.9300
C9—C10	1.380 (5)	C26—C27	1.359 (6)
C10—C11	1.388 (6)	C26—H26	0.9300
C10—H10	0.9300	C27—C28	1.369 (5)
C11—C12	1.361 (6)	C27—C30	1.513 (6)
C11—H11	0.9300	C28—C29	1.376 (5)
C12—C13	1.376 (5)	C28—H28	0.9300
C12—H12	0.9300	C29—H29	0.9300
C13—C14	1.371 (5)	C30—H30A	0.9600
C13—H13	0.9300	C30—H30B	0.9600
C15—N2	1.470 (4)	C30—H30C	0.9600
			
N1—C1—C6	111.1 (3)	N2—C15—H15B	109.2
N1—C1—C2	111.6 (2)	C16—C15—H15B	109.2
C6—C1—C2	112.5 (3)	H15A—C15—H15B	107.9
N1—C1—H1	107.1	C17—C16—C21	116.2 (4)
C6—C1—H1	107.1	C17—C16—C15	122.0 (4)
C2—C1—H1	107.1	C21—C16—C15	121.8 (3)
C3—C2—C1	112.1 (3)	C16—C17—C18	121.2 (4)
C3—C2—H2A	109.2	C16—C17—H17	119.4
C1—C2—H2A	109.2	C18—C17—H17	119.4
C3—C2—H2B	109.2	C19—C18—C17	121.9 (4)
C1—C2—H2B	109.2	C19—C18—H18	119.0
H2A—C2—H2B	107.9	C17—C18—H18	119.0
C4—C3—C2	111.0 (3)	C18—C19—C20	117.2 (4)
C4—C3—H3A	109.4	C18—C19—C22	122.0 (4)
C2—C3—H3A	109.4	C20—C19—C22	120.8 (4)
C4—C3—H3B	109.4	C19—C20—C21	121.3 (4)
C2—C3—H3B	109.4	C19—C20—H20	119.3
H3A—C3—H3B	108.0	C21—C20—H20	119.3
C3—C4—C5	110.3 (3)	C20—C21—C16	122.2 (4)
C3—C4—H4A	109.6	C20—C21—H21	118.9
C5—C4—H4A	109.6	C16—C21—H21	118.9
C3—C4—H4B	109.6	C19—C22—H22A	109.5
C5—C4—H4B	109.6	C19—C22—H22B	109.5
H4A—C4—H4B	108.1	H22A—C22—H22B	109.5
C4—C5—C6	112.5 (3)	C19—C22—H22C	109.5
C4—C5—H5A	109.1	H22A—C22—H22C	109.5
C6—C5—H5A	109.1	H22B—C22—H22C	109.5
C4—C5—H5B	109.1	N2—C23—C24	111.9 (2)
C6—C5—H5B	109.1	N2—C23—H23A	109.2
H5A—C5—H5B	107.8	C24—C23—H23A	109.2
N2—C6—C5	114.9 (2)	N2—C23—H23B	109.2
N2—C6—C1	112.5 (3)	C24—C23—H23B	109.2
C5—C6—C1	109.2 (3)	H23A—C23—H23B	107.9
N2—C6—H6	106.6	C29—C24—C25	118.0 (3)
C5—C6—H6	106.6	C29—C24—C23	120.4 (3)
C1—C6—H6	106.6	C25—C24—C23	121.5 (3)
O1—C7—N1	124.5 (3)	C24—C25—C26	119.7 (4)
O1—C7—C14	128.7 (3)	C24—C25—H25	120.2
N1—C7—C14	106.8 (2)	C26—C25—H25	120.2
O2—C8—N1	125.6 (3)	C27—C26—C25	122.2 (3)
O2—C8—C9	128.6 (3)	C27—C26—H26	118.9
N1—C8—C9	105.8 (2)	C25—C26—H26	118.9
C14—C9—C10	121.1 (3)	C26—C27—C28	117.5 (4)
C14—C9—C8	108.5 (3)	C26—C27—C30	121.4 (4)
C10—C9—C8	130.4 (3)	C28—C27—C30	121.1 (5)
C9—C10—C11	117.3 (3)	C27—C28—C29	121.1 (4)
C9—C10—H10	121.3	C27—C28—H28	119.4
C11—C10—H10	121.3	C29—C28—H28	119.4
C12—C11—C10	120.8 (4)	C24—C29—C28	121.4 (4)
C12—C11—H11	119.6	C24—C29—H29	119.3
C10—C11—H11	119.6	C28—C29—H29	119.3
C11—C12—C13	121.7 (4)	C27—C30—H30A	109.5
C11—C12—H12	119.1	C27—C30—H30B	109.5
C13—C12—H12	119.1	H30A—C30—H30B	109.5
C14—C13—C12	117.5 (3)	C27—C30—H30C	109.5
C14—C13—H13	121.2	H30A—C30—H30C	109.5
C12—C13—H13	121.2	H30B—C30—H30C	109.5
C9—C14—C13	121.4 (3)	C7—N1—C8	110.9 (3)
C9—C14—C7	108.0 (3)	C7—N1—C1	123.1 (2)
C13—C14—C7	130.7 (3)	C8—N1—C1	125.8 (3)
N2—C15—C16	112.1 (3)	C6—N2—C23	111.6 (3)
N2—C15—H15A	109.2	C6—N2—C15	113.9 (3)
C16—C15—H15A	109.2	C23—N2—C15	111.1 (2)
			
N1—C1—C2—C3	−179.3 (3)	C18—C19—C20—C21	−1.0 (7)
C6—C1—C2—C3	−53.7 (4)	C22—C19—C20—C21	179.3 (4)
C1—C2—C3—C4	54.5 (5)	C19—C20—C21—C16	1.1 (8)
C2—C3—C4—C5	−56.2 (5)	C17—C16—C21—C20	0.1 (7)
C3—C4—C5—C6	58.2 (5)	C15—C16—C21—C20	−178.3 (4)
C4—C5—C6—N2	176.5 (3)	N2—C23—C24—C29	−61.9 (4)
C4—C5—C6—C1	−56.0 (4)	N2—C23—C24—C25	119.8 (3)
N1—C1—C6—N2	−52.1 (3)	C29—C24—C25—C26	−1.6 (5)
C2—C1—C6—N2	−178.0 (3)	C23—C24—C25—C26	176.9 (3)
N1—C1—C6—C5	179.1 (3)	C24—C25—C26—C27	0.6 (5)
C2—C1—C6—C5	53.2 (4)	C25—C26—C27—C28	0.6 (6)
O2—C8—C9—C14	178.8 (3)	C25—C26—C27—C30	−179.6 (4)
N1—C8—C9—C14	−0.7 (3)	C26—C27—C28—C29	−0.8 (6)
O2—C8—C9—C10	−1.2 (6)	C30—C27—C28—C29	179.4 (4)
N1—C8—C9—C10	179.3 (3)	C25—C24—C29—C28	1.4 (5)
C14—C9—C10—C11	−0.6 (5)	C23—C24—C29—C28	−177.1 (3)
C8—C9—C10—C11	179.4 (3)	C27—C28—C29—C24	−0.2 (6)
C9—C10—C11—C12	0.7 (6)	O1—C7—N1—C8	−179.6 (3)
C10—C11—C12—C13	−1.2 (7)	C14—C7—N1—C8	−0.6 (3)
C11—C12—C13—C14	1.5 (6)	O1—C7—N1—C1	5.5 (4)
C10—C9—C14—C13	1.0 (5)	C14—C7—N1—C1	−175.5 (2)
C8—C9—C14—C13	−179.0 (3)	O2—C8—N1—C7	−178.7 (3)
C10—C9—C14—C7	−179.6 (3)	C9—C8—N1—C7	0.8 (3)
C8—C9—C14—C7	0.4 (3)	O2—C8—N1—C1	−4.0 (5)
C12—C13—C14—C9	−1.4 (5)	C9—C8—N1—C1	175.5 (3)
C12—C13—C14—C7	179.4 (3)	C6—C1—N1—C7	108.9 (3)
O1—C7—C14—C9	179.1 (3)	C2—C1—N1—C7	−124.7 (3)
N1—C7—C14—C9	0.1 (3)	C6—C1—N1—C8	−65.2 (4)
O1—C7—C14—C13	−1.6 (5)	C2—C1—N1—C8	61.2 (4)
N1—C7—C14—C13	179.5 (3)	C5—C6—N2—C23	−82.1 (4)
N2—C15—C16—C17	120.1 (3)	C1—C6—N2—C23	152.1 (3)
N2—C15—C16—C21	−61.6 (5)	C5—C6—N2—C15	44.6 (4)
C21—C16—C17—C18	−1.3 (6)	C1—C6—N2—C15	−81.2 (3)
C15—C16—C17—C18	177.1 (3)	C24—C23—N2—C6	−73.6 (4)
C16—C17—C18—C19	1.3 (6)	C24—C23—N2—C15	158.1 (3)
C17—C18—C19—C20	−0.1 (6)	C16—C15—N2—C6	164.1 (3)
C17—C18—C19—C22	179.5 (4)	C16—C15—N2—C23	−68.9 (4)

## References

[bb1] Bruker (2000). *SMART* and *SAINT* Bruker AXS Inc., Madison, Wisconsin, USA.

[bb2] Gawronski, J., Kazmierczak, F., Gawronska, K., Rychlewska, U., Nordén, B. & Holmén, A. (1998). *J. Am. Chem. Soc.* **120**, 12083–12091.

[bb3] Kaik, M. & Gawroński, J. (2003). *Tetrahedron Asymmetry*, **14**, 1559–1563.

[bb4] Paradowska, J., Rogozinóska, M. & Mlynarski, J. (2009). *Tetrahedron Lett.* **50**, 1639–1641.

[bb5] Sheldrick, G. M. (2008). *Acta Cryst.* A**64**, 112–122. 10.1107/S0108767307043930 18156677

